# Highly pathogenic avian influenza virus H5N2 (clade 2.3.4.4) challenge of mallards age appropriate to the 2015 midwestern poultry outbreak

**DOI:** 10.1111/irv.12886

**Published:** 2021-07-29

**Authors:** Jeffrey S. Hall, Daniel A. Grear, Scott Krauss, J. Patrick Seiler, Robert J. Dusek, Sean W. Nashold, Robert G. Webster

**Affiliations:** ^1^ United States Geological Survey, National Wildlife Health Center Madison WI USA; ^2^ Infectious Disease Department St. Jude Children's Research Hospital Memphis TN USA

**Keywords:** adult, highly pathogenic, influenza, mallards, outbreak, poultry

## Abstract

**Background:**

The 2015 highly pathogenic avian influenza virus (HPAIV) H5N2 clade 2.3.4.4 outbreak in upper midwestern U.S. poultry operations was not detected in wild birds to any great degree during the outbreak, despite wild waterfowl being implicated in the introduction, reassortment, and movement of the virus into North America from Asia. This outbreak led to the demise of over 50 million domestic birds and occurred mainly during the northward spring migration of adult avian populations.

**Objectives:**

There have been no experimental examinations of the pathogenesis, transmission, and population impacts of this virus in adult wild waterfowl with varying exposure histories—the most relevant age class.

**Methods:**

We captured, housed, and challenged adult wild mallards (*Anas platyrhynchos*) with HPAIV H5N2 clade 2.3.4.4 and measured viral infection, viral excretion, and transmission to other mallards.

**Results:**

All inoculated birds became infected and excreted moderate amounts of virus, primarily orally, for up to 14 days. Cohoused, uninoculated birds also all became infected. Serological status had no effect on susceptibility. There were no obvious clinical signs of disease, and all birds survived to the end of the study (14 days).

**Conclusions:**

Based on these results, adult mallards are viable hosts of HPAIV H5N2 regardless of prior exposure history and are capable of transporting the virus over short and long distances. These findings have implications for surveillance efforts. The capture and sampling of wild waterfowl in the spring, when most surveillance programs are not operating, are important to consider in the design of future HPAIV surveillance programs.

## 1 INTRODUCTION

In November/December 2014, highly pathogenic avian influenza virus (HPAIV) H5N8 was discovered in British Columbia, Canada and the nearby state of Washington, USA.^1^, ^2^ This was from the Eurasian (EA) H5 clade 2.3.4.4 that originated in China and spread by waterfowl across Asia into Europe in 2014.^3^ It was the first detection of this H5 clade 2.3.4.4 HPAIV in North America (NA) and it was likely transported into NA by migrating waterfowl from Asia through Alaska and the Pacific flyway.^4^, ^5^ The HPAIV quickly reassorted with low pathogenic avian influenza viruses (LPAIVs) that are essentially indigenous in NA waterfowl, into hybrid EA/NA HPAIV including H5N1 and H5N2 subtypes.^6^, ^7^ These viruses were subsequently detected in wild bird and poultry surveillance efforts throughout the Pacific flyway and sporadic locations elsewhere in the western United States.^8^

In early March 2015, the HPAIV H5N2 clade 2.3.4.4A virus appeared in a Minnesota poultry operation and over the next 3–4 months led to the destruction of more than 50 million birds at 110 facilities in five states, in efforts to stem the outbreak. The economic cost of the outbreak and control measures was estimated to be nearly 5 billion dollars.^9^

The roles of wild waterfowl in the transport and transmission of this virus during the outbreak are unknown. No HPAIV was detected in wild waterfowl in the poultry outbreak area, and the only wild birds found with the virus in that region were a black‐capped chickadee (*Poecile atricapillus*) and a Cooper's hawk (*Accipiter cooperii*) in Minnesota and a Wisconsin snowy owl (*Nyctea scandiaca*). These detections all came from passive collection of moribund or dead birds and raise the issue of how efficient and effective surveillance in wild birds was during this agricultural emergency.^10^

To better define how wild waterfowl were involved in the outbreak, we challenged adult wild mallards (*Anas platyrhynchos*), as well as 8‐month‐old mallards, with the HPAIV H5N2 clade 2.3.4.4A virus from the outbreak. These were the ages of birds that coincided with the ages of wild waterfowl present during the outbreak—adult birds with histories of previous LPAIV exposure and younger birds hatched the previous summer. We measured infection, virus excretion, and transmission and documented any morbidity and health effects of the HPAIV infection in these birds. We specifically examined whether age and previous exposure to LPAIVs impacted the timing and intensity of viral shedding, as well as the transmissibility to naïve birds. Knowledge of HPAIV ecology in wild waterfowl is critical for developing effective surveillance systems, biosecurity measures, and meaningful risk analyses.

## 2 METHODS

### 2.1 Mallard acquisition and care

Twenty‐seven adult wild mallards (after hatch year), both male and female, were captured using rocket nets at Horicon National Wildlife Refuge, Wisconsin (latitude: 43.570484, longitude: −88.608035) in August 2018 during seasonal banding operations. The birds were transported to the USGS National Wildlife Health Center (NWHC), Madison, Wisconsin and group housed in a BSL‐3 biocontainment animal room. Thirteen hatchling mallards were purchased from a commercial source in September 2018 (Murray McMurray Hatchery, Webster City, IA) and brooded and housed in a separate BSL‐3 room from the adult birds to prevent naturally acquired infections being transmitted from the adults to the immunologically naïve young birds. All birds had their primary flight feathers clipped, were provided food and water ad libitum, and allowed free movement within the room with access to tubs of fresh water for bathing and swimming. All husbandry and experimental procedures were performed according to methods approved by the NWHC Institutional Animal Care and Use Committee. Birds were housed in BSL‐3 for ~8 months until the commencement of the study in April 2019. Prior to start of the study, the birds were moved into high efficiency particulate air (HEPA)‐filtered isolator cages (two birds/cage) with a mixture of seropositive adults, seronegative adults, and young birds (Table 1). The birds acclimated in the cages for 3 days prior to the commencement of the study.

**TABLE 1:** Experimental setup of mallard HPAIV H5N2 clade 2.3.4.4A challenge

Cage number	Bird ID^a^	Age^b^	Preinoculation serological status^c^	Treatment
1	R45^d^	Adult	Negative	Mock inoculation
	264B^d^	8 MO	Negative	Mock inoculation
2	R100	Adult	Positive	HPAIV inoculation
	R395	Adult	Negative	Contact transmission
3	R66	Adult	Negative	HPAIV inoculation
	Y186	Adult	Negative	Contact transmission
4	R41	Adult	Positive	HPAIV inoculation
	R42	Adult	Negative	Contact transmission
5	Y268	Adult	Positive	HPAIV inoculation
	Y269	Adult	Negative	Contact transmission
6	Y298	Adult	Positive	HPAIV inoculation
	Y189	Adult	Positive	Contact transmission
7	Y293	Adult	Positive	HPAIV inoculation
	Y285	Adult	Negative	Contact transmission
8	R35	Adult	Positive	HPAIV inoculation
	R48	Adult	Positive	Contact transmission
9	Y271	Adult	Negative	HPAIV inoculation
	Y279	Adult	Positive	Contact transmission
10	Y257	Adult	Negative	HPAIV inoculation
	Y251	Adult	Positive	Contact transmission
11	Y252B	8 MO	Negative	HPAIV inoculation
	Y261	Adult	Negative	Contact transmission
12	Y265B	8 MO	Negative	HPAIV inoculation
	Y286	Adult	Negative	Contact transmission
13	Y257B	8 MO	Negative	HPAIV inoculation
	R498	Adult	Positive	Contact transmission
14	R43	Adult	Positive	HPAIV inoculation
	Y262B	8 MO	Negative	Contact transmission
15	Y297	Adult	Negative	HPAIV inoculation
	Y261B	8 MO	Negative	Contact transmission
16	R46	Adult	Negative	HPAIV inoculation
	Y260B	8 MO	Negative	Contact transmission
17	R369	Adult	Positive	HPAIV inoculation
	Y259B	8 MO	Negative	Contact transmission
18	Y290	Adult	Negative	HPAIV inoculation
	Y251B	8 MO	Negative	Contact transmission
19	Y254B	8 MO	Negative	HPAIV inoculation
	Y256B	8 MO	Negative	Contact transmission
20	Y258B	8 MO	Negative	HPAIV inoculation
	Y263B	8 MO	Negative	Contact transmission

^a^ 8‐month‐old mallards designated with B after the ID number.

^b^ Adults were ≥2 years old (after hatch year).

^c^ Serological status at time of inoculation (DPI 0) as determined by IDEXX ELISA.

^d^ Mock‐inoculated control.

Abbreviation: HPAIV, highly pathogenic avian influenza virus.

### 2.2 Serological testing

Blood was collected from the wild‐caught adult mallards by jugular venipuncture in November 2018 and from all birds in May 2019. These were 6 months before and immediately prior to initiation of the study, respectively. Sera were separated from the cellular blood components by centrifugation and stored at −20°C until tested for influenza virus antibodies using the MultiS‐Screen AI Virus Antibody Kit (IDEXX Laboratories Westbrook, ME) according to the manufacturer's instructions. This assay detects antibodies to influenza A virus (IAV) nucleoprotein (NP).

Hemagglutination inhibition (HI) assays were performed on a panel of reference viruses representing IAV HA subtypes frequently identified in wild waterfowl.^11^ Sera were receptor‐destroying enzyme (RDE) treated (Seiken) according to manufacturer's instructions. Sera were heat treated at 56°C for 30 min, and phosphate‐buffered saline (PBS) was added to dilute the sera to 1:10. We titrated the sera using serial twofold dilutions in 96‐well microtiter plates leaving 25‐μl serum dilution in each well. Virus antigens were diluted to four hemagglutinating units, 25 μl added to the sera dilutions, and incubated 1 h at room temperature; 50‐μl 0.5% chicken red blood cells were added to each well and incubated for 30 min, when HI reactions were read. In a separate assay, we used horse red blood cells instead of chicken red blood cells following the same procedure. Table S1 lists the IAV used in the HI assays.

### 2.3 Virus

The HPAIV used to inoculate the birds, A/Turkey/MN/11668‐1/2015 (H5N2) clade 2.3.4.4A, was provided by St. Jude Children's Research Hospital. This is the index virus from the poultry outbreak in the Midwest United States and was passaged twice in embryonated chicken eggs.

### 2.4 Inoculation and sampling

The virus inoculum was prepared by diluting the stock virus isolate in brain heart infusion (BHI). The inoculum virus titer was confirmed in embryonated chicken eggs according to the method of Reed and Muench.^12^ One bird in each cage was inoculated intrachoanally with 10^5^ 50% egg infectious doses (EID_50_) of virus in 1 ml of BHI using a 1‐ml syringe tipped with a metal canula. The birds in cage #1, one adult and one young bird, were mock inoculated using a corresponding volume of BHI. The experimental design is outlined in Table 1. All birds were sampled prior to inoculation and every second day postinoculation (DPI) until DPI 14 when the study was terminated, the birds were humanely euthanized, and final blood samples were collected by cardiac puncture. Sampling consisted of obtaining separate cloacal and oropharyngeal swabs using Dacron tipped applicators and placed in cryovials containing 1‐ml viral transport media (Hanks Balanced Salt Solution, 0.05% gelatin, 5% glycerin, 1500 units/ml penicillin, 1500 mg/ml streptomycin, 0.1 mg/ml gentamicin, 1 mg/ml fungizone) and stored at −80°C until analyses. All birds were monitored at least twice daily and weighed at each sampling to monitor health status.

### 2.5 Quantitative reverse transcription‐polymerase chain reaction

Viral RNA was extracted from cloacal and oropharyngeal swabs using the MagMAXTM‐96 AI/ND Viral RNA Isolation Kit (Applied Biosystems, Foster City, CA) following the manufacturer's procedures. Real‐time reverse transcription‐polymerase chain reaction (RT‐PCR) was performed using the published procedure of Spackman et al.^13^ qRT‐PCR assays used reagents provided in the Qiagen OneStep RT‐PCR kit (Qiagen, Hilden, Germany) and performed on a Stratagene Mx3005P thermal cycler (San Diego, CA). *C*_t_ values of duck swabs were compared with those from a standard curve of known viral concentrations to calculate the amounts of virus excreted by infected ducks and were reported as EID_50_ RNA equivalents/ml.^14^ On the basis of serial dilutions of the virus and the standard curve, we estimated the limit of detection of these methods to be between 0 and 10 EID_50_ RNA equivalents/ml.

### 2.6 Analysis of infection dynamics and transmission

We assessed the transmissibility of HPAIV H5N2 clade 2.3.4.4A by comparing the proportion of noninoculated cage mates with detectable viral RNA from oral swabs before 14 DPI between ages and serostatus. We used a nonlinear hierarchical model to estimate the viral shedding dynamics of the inoculated birds. This analytical method allowed us to estimate key mechanistic parameters that control transmission through viral shedding intensity and timing. The response data (EID_50_ equivalents) were fit to a nonlinear function that has been demonstrated to capture the shape of viral infection dynamics in AIV challenges,^15^, ^16^

$${\mathrm{EID}}_{t}=\frac{2\left(V_{p}+\beta_{v,1-2}\right)}{e^{-g*\left(t-\left(t_{p+}\beta_{t_{p},1-2}\right)\right)}+e^{\left(d+\beta_{d,1-2}\right)*\left(t-\left(t_{p+}\beta_{t_{p},1-2}\right)\right)}}.$$

*V*_*p*_ was the maximum EID_50_ excretion at the infection peak for mallards that were seropositive for IAV exposure on the inoculation day with coefficients *β*_*v*,1 − 2_ estimated for effect sizes of seronegative adults (*β*_,1_) and subadults (*β*_2_); *t*_*p*_ was the estimate of the time (days postinfection) of the maximum EID_50_, and $\left(\beta_{t_{p},1-2}\right)$ were the estimated effect sizes of adult and subadult maximum EID_50_; *g* was the exponential growth rate of viral shedding, *d* was the exponential rate of viral shedding decline, (*β*_*d*,1 − 2_) were the estimates of decline effect sizes of adults and subadults, and *t* was the time (DPI) of the observed EID_50_ response. We modeled the EID_50_ response as a lognormally distributed response variable with hierarchical components to incorporate individual variation using Bayesian implementation and Gibbs sampling to estimate the mean values for *V*_p_, *t*_p_, and *d*, as well as coefficients for effects of seronegative adults ( $\beta_{v,1},\beta_{t_{p},1},\beta_{d,1}$) and 8‐month‐old birds ( $\beta_{v,2},\beta_{t_{p},2},\beta_{d,2}$). We did not estimate the exponential growth rate parameter, *g*, because it was nonidentifiable given the sampling frequency. Rather, we included this as an informative closed‐form prior that was bounded by biologically relevant values.^17^ We did not attempt to model the cloacal shedding dynamics because the detection through the time was much more variable than oral detection. Details on the model, selection of priors, and fit evaluation are presented in Appendix A.

## 3 RESULTS

### 3.1 Preexposure serology

We tested adult mallards for the presence of avian influenza antibodies using a commercial enzyme‐linked immunosorbent assay (ELISA) that detects antibodies to the viral NP. This assay is extensively used to measure the serological status of wild ducks.^18^ In the November sampling, 16/28 adult mallards were positive for the presence of IAV antibodies (Table 2). Six months later, just prior to the initiation of the challenge, 14/28 remained seropositive by ELISA. The two that became seronegative over this timeframe became only marginally negative so the serostatus of the adult mallards remained relatively stable over the 8+ months that they were housed in biocontainment. The 8‐month‐old mallards all remained seronegative prior to the commencement of the study (data not shown).

**TABLE 2:** Serological status of adult mallards at two time points before and postinoculation with highly pathogenic avian influenza virus H5N2 clade 2.3.4.4A

Bird ID	Nov. 2018 (S/N)^a^	May 2019^b^ (S/N)	Postchallenge^c^ (S/N)
R45^d^	Negative (0.674)	Negative (0.598)	Negative (0.650)
R100	Positive (0.366)	Positive (0.467)	Negative (0.610)
R395	Positive (0.499)	Negative (0.546)	Positive (0.166)
R66	Positive (0.161)	Positive (0.120)	Positive (0.091)
Y186	Negative (0.692)	Negative (0.662)	Positive (0.096)
R41	Positive (0.06)	Positive (0.068)	Positive (0.071)
R42	Negative (0.721)	Negative (0.885)	Positive (0.080)
Y268	Positive (0.442)	Positive (0.384)	Positive (0.321)
Y269	Negative (0.660)	Negative (0.611)	Positive (0.055)
Y298	Positive (0.428)	Positive (0.474)	Positive (0.114)
Y189	Positive (0.086)	Positive (0.084)	Positive (0.087)
Y293	Positive (0.217)	Positive (0.277)	Positive (0.249)
Y285	Negative (0.860)	Negative (0.797)	Positive (0.085)
R35	Positive (0.138)	Positive (0.150)	Positive (0.164)
R48	Positive (0.089)	Positive (0.087)	Positive (0.154)
Y271	Negative (0.514)	Negative (0.538)	Positive (0.086)
Y279	Positive (0.079)	Positive (0.077)	Positive (0.084)
Y257	Negative (0.510)	Negative (0.544)	Positive (0.164)
Y251	Positive (0.469)	Positive (0.427)	Positive (0.100)
Y261	Negative (0.609)	Negative (0.536)	Positive (0.091)
Y286	Negative (0.713)	Negative (0.845)	Positive (0.144)
R498	Positive (0.379)	Positive (0.436)	Positive (0.090)
R43	Positive (0.257)	Positive (0.208)	Positive (0.093)
Y297	Negative (3.095)	Negative (0.736)	Positive (0.208)
R46	Negative (0.624)	Negative (0.587)	Positive (0.089)
R369	Positive (0.087)	Positive (0.110)	Positive (0.069)
Y290	Positive (0.481)	Negative (0.523)	Positive (0.089)

*Note*: Sera were tested with IDEXX MultiS‐Screen AI Virus Antibody ELISA Kit.

^a^ S/N ratio = sample mean absorbance_(650)_/negative control mean absorbance_(650)_. S/N ratios <0.50 are considered positive.

^b^ Sera collected one day prior to inoculation with highly pathogenic avian influenza virus H5N2 clade 2.3.4.4A.

^c^ Sera collected at end of study (14‐day postinoculation).

^d^ Mock‐inoculated control.

To characterize the virus exposure histories of the seropositive birds, we performed HI analyses on the adult mallard sera using an array of virus subtypes common in wild waterfowl, as well as the H5N8 and H5N2 HPAI clade 2.3.4.4A viruses from 2014/2015. None of the sera inhibited hemagglutination from any of the viruses utilized except the sera from duck Y290 reacted weakly (1:10 dilution) to the H4 isolate, and duck Y271 also reacted at 1:10 to the LPAIV H5N2 isolate. None of the sera contained preexisting antibodies to the HPAIV tested.

### 3.2 HPAIV infection, excretion, and transmission in mallards

**FIGURE 1:**
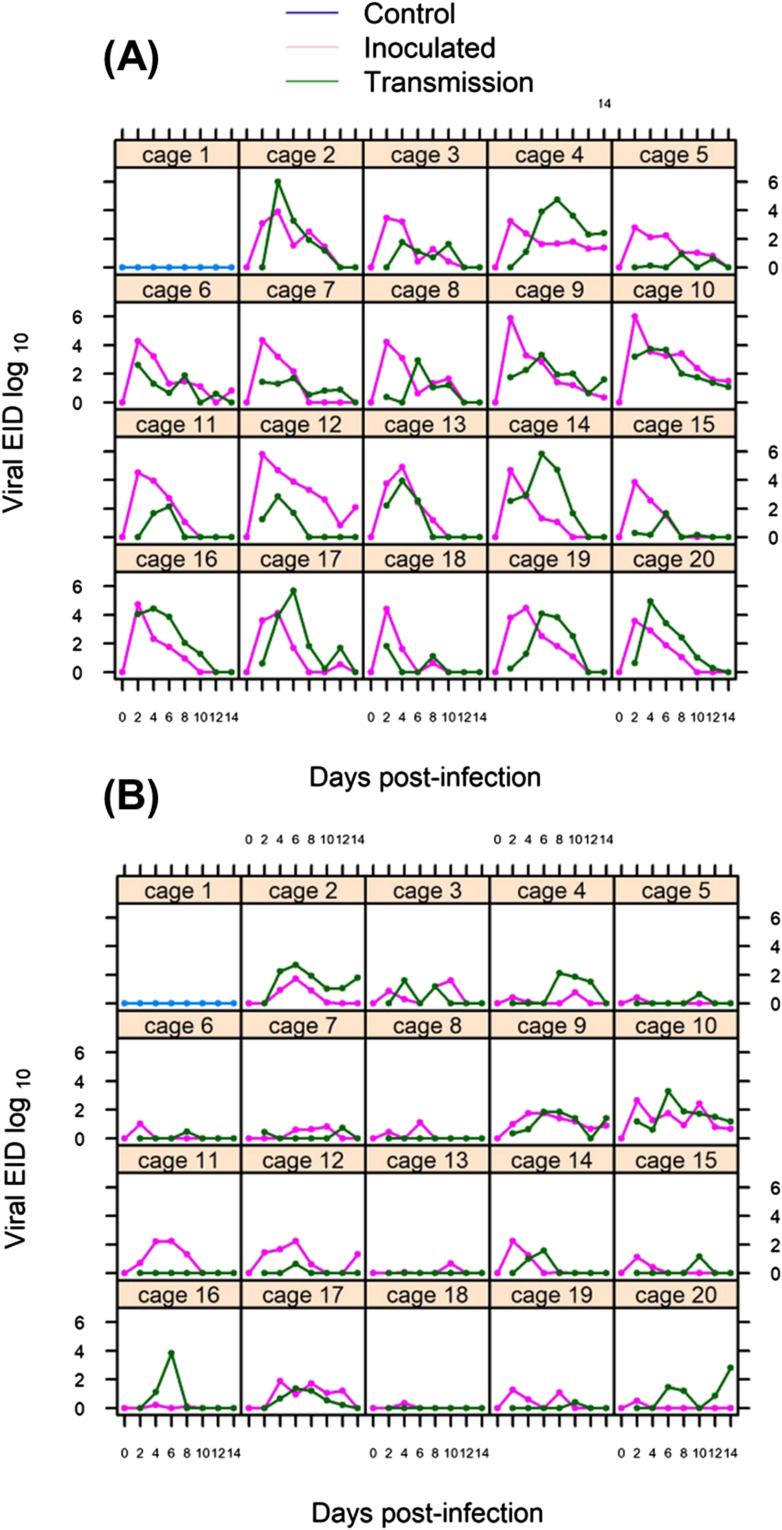
Viral excretion measured in egg infectious doses (EID_50_) RNA equivalents/ml, log scale via oral shedding (A) and cloacal shedding (B). Inoculated birds (purple lines) were intrachoanally inoculated with 10^5^ EID_50_ on Day 0 and placed in a cage with one noninoculated bird to measure transmission (green lines). Each panel represents one cage with one pair of ducks (see Table 1). The limit of detection was 0–10 EID_50_ RNA equivalents

Following intrachoanal inoculation with the HPAIV, all inoculated birds became infected and excreted detectable virus by DPI2 (Figure 1A). Neither the serological status nor the age of the birds had detectable effects on intensity or timing of oral virus excretion. Maximum oral viral shedding in adult mallards with previous exposure (mean log EID_50_ and 95% credible interval [CI]; *V*_*p*_ = 8.60 [6.3, 12.1]), effect size in adult mallards with no prior exposure (*β*_*v*,1_ = 0.16 [−1.2, 2.2]), and effect size in 8 month‐old‐mallards (*β*_*v*,2_ = 0.01 [−1.6, 1.6]). Time to maximum viral shedding in adult mallards with serologic exposure, *t*_*p*_ = 1.9 days [1.2, 2.2], effect size in adult mallards with no serological exposure $(\beta_{t_{p},1}$ = − 0.04 [−0.5, 0.4]), and effect size in 8‐month‐old mallards $(\beta_{t_{p},2}$ = 0.24 [−1.0, 0.2]). All noninoculated birds cohoused with an inoculated bird became infected by DPI 2 or 4, with viral detection via oral excretion in 19/19 and detection via cloacal shedding in 15/19 (Figure 1). The predominate route of excretion in all birds was oral with lesser amounts of virus excreted cloacally.

The duration of the infections varied from the shortest ending at 6 DPI to longer than 14 DPI with no difference in decay rate of oral shedding detected in inoculated birds (*d* = 0.5 [0.32,0.73],*β*_*d*,1_ =  − 0.13 [−0.33,0.06],*β*_*d*,2_ =  − 0.03 [−0.26,0.22]). Interestingly, eight birds, some inoculated and some transmission subjects, remained infected, based on virus shedding, for the entire 14 days of the study (Figure 1). Thus, we were unable to estimate an accurate endpoint of the infection process.

### 3.3 Disease signs in HPAIV infected mallards

All mallards survived till the end of this study except one bird, Y252B (Cage 11), that was euthanized on DPI 12 for overly aggressive behavior towards its cagemate. None of the infected birds, either by inoculation or transmission, or the control birds showed any overt clinical signs of disease over the course of this study. Most of the ducks, including the control birds, showed moderate loss of weight (6.8% mean, Table S2) by DPI 4; however, this weight was typically regained by DPI 10 and was possibly a response to the stress of being placed in the isolator cages and handling during sampling.

## 4 DISCUSSION

We experimentally challenged adult wild mallards and young birds raised in biocontainment with the HPAIV H5N2 clade 2.3.4.4A virus that led to the deaths of millions of midwestern poultry in the first half of 2015.^9^ These ages coincided with those of wild waterfowl that were present at the time of the outbreak, adults with histories of exposure to influenza viruses and young birds hatched the previous summer. Interestingly, 16 of the wild‐source adult birds had detectable antibodies when first tested based on the NP ELISA. Fourteen of those remained seropositive 6 months later at the time of the study, yet only two of those were weakly positive (1:10) by HI assay. The differences between these two assays are important to consider. The competitive ELISA measures the amount of antibody to the NP, an internal protein in the influenza virion. It is not known if these antibodies are more stable than antibodies to the viral hemagglutinin (HA) protein that are measured by HI or if the assay is just that much more sensitive. We measured a relatively stable antibody persistence of at least 8 months from the latest possibility of exposure to IAV prior to capture, until the preinoculation sampling. There was no evidence any of the birds had exposure to the clade 2.3.4.4 HPAIVs that were related to the source of our inoculum.

The lifespan of duck antibodies is nebulous. Curran et al.^19^ reported similar findings with NP‐ELISA results being stable for 8 months in wild birds while their HI results declined within 56 days. In contrast, other researchers reported declining ELISA readings over 28 days while the HI results remained stable over that span.^20^ Both assays are routinely used by researchers, and the differences in the results are an important consideration. The stability of a duck's serological response to infection is a critical component to understand for accurate risk analyses and epidemiological studies of HPAIV outbreaks and the ecology of IAV in natural systems. Additional long‐term experimental studies on this topic are warranted. Regardless, any antibodies present in our subjects at the time of the study, including the bird with a weak LPAIV H5N2 HI titer, had no effect on infection, shedding dynamics, or transmission of HPAIV.

All exposed mallards in our study became infected with the HPAIV, either by direct inoculation or by transmission. There were no clinical signs or indications of disease in any of the infected mallards, similar to findings from other experimental challenge studies.^21^, ^22^, ^23^, ^24^, ^25^ These studies are typically conducted with very young, immunologically naïve birds as test subjects. We were the first to capture, house, and challenge adult wild ducks that had a variety of IAV exposure histories, in comparison with young, 8‐month‐old, naïve birds. Adult birds comprised the waterfowl population migrating through and inhabiting the region of the HPAIV outbreak during the spring of 2015 and were the most appropriate subjects for this study to provide relevant data for wild waterfowl during the outbreak.

This HPAIV readily infects adult wild ducks with no obvious ill effects, with moderate viral excretion and transmission. Thus, HPAIV H5N2 clade 2.3.4.4A infection in these birds basically behaves similar to infection with LPAIV, which are ubiquitous in wild duck populations.^26^ Consequently, as confirmed by our modeling, wild ducks can serve as natural hosts, have no transmission barriers to persistence, and can be involved in the transport of the HPAIV over short and long distances.^27^, ^28^ The hypothesized mechanisms of the multiple virus introductions and spread within and between poultry operations are varied, including being downwind of infected sites, anthropogenic methods including carcass and garbage movement and disposal, and lax biosecurity measures.^29^, ^30^ None of these would preclude infection of wild populations and in fact would be just as likely to spread the virus to those populations as they would to domestic poultry.

Given the massive, widespread outbreak in poultry, why did not the large population of wild ducks in the region become infected with the HPAIV to the point that surveillance systems could detect it? In contrast to widespread detection of HPAIV in hunter killed waterfowl in the Pacific flyway, none of the limited active surveillance conducted during the poultry outbreak found evidence of HPAIV H5N2 clade 2.3.4.4A in any wild duck.^31^, ^32^, ^33^, ^34^ In fact, the only active surveillance evidence was detection of HPAI viral RNA from a single European starling (*Sturnus vulgaris*) and serological evidence from several American robins (*Turdus migratorius*) captured and sampled at a poultry facility during an active outbreak.^35^ The only other documented findings of HPAIV H5N2 clade 2.3.4.4A from wild birds in the outbreak area were from a Cooper's Hawk, a snowy owl, and a black‐capped chickadee, all collected due to severe morbidity and/or mortality. Several additional reports, based on morbidity/mortality, of HPAIV H5N2 clade 2.3.4.4A infection in Canada geese (*Branta canadensis*) occurred outside of the outbreak region.^10^

Wild waterfowl were clearly instrumental in the introduction, reassortment, and dissemination of HPAIV H5 viruses into NA in 2014 and 2015. Even though the HPAIV H5N2 clade 2.3.4.4A virus was isolated from poultry, our findings show it readily infects all ages of mallards and underscores the potential that the virus could still circulate in wild duck populations undetected by the limited surveillance efforts. Since the outbreak there has been only one detection of HPAIV H5N2 clade 2.3.4.4A in wild waterfowl, from a single mallard sampled in Alaska in the summer of 2016.^36^ Traditionally, active IAV surveillance in waterfowl occurs primarily in the autumn when large numbers of birds are routinely captured and sampled at premigratory congregation sites and migratory stopover locations and by opportunistic sampling of hunter harvested birds.^37^ None of these methods of capture and sampling were readily available in the spring of 2015 during the outbreak, so unfortunately, there are few data available to determine the virus occurrence or prevalence in wild duck populations. Using passive surveillance in northward migrating wild ducks as the preferential means of detection of HPAIV, particularly because the virus causes no clinical signs, morbidity or mortality in these birds severely limits the likelihood of providing early warning and monitoring of potential outbreaks.^35^ This is important and must be considered in the design of future surveillance efforts.

## AUTHOR CONTRIBUTIONS

**Daniel A. Grear:** Data curation; formal analysis; methodology. **Scott Krauss:** Conceptualization; investigation; methodology. **Patrick Seiler:** Data curation; formal analysis; investigation; methodology. **Robert J. Dusek:** Investigation; methodology. **Sean W. Nashold:** Data curation; formal analysis; investigation; methodology. **Robert G. Webster:** Conceptualization; investigation; methodology.

## CONFLICT OF INTEREST

None of the authors have any financial interests or conflicts of interest. Use of product, trade, or firm names is for descriptive purposes only and does not imply endorsement by the U. S. government.

## Supporting information


**Table S1.** Avian influenza virus isolates used in hemagglutination inhibition assays.

**Table S2.** Body weights (grams) of highly pathogenic avian influenza virus H5N2 clade 2.3.4.4A challenged mallards

## Figures and Tables

**FIGURE A1:**
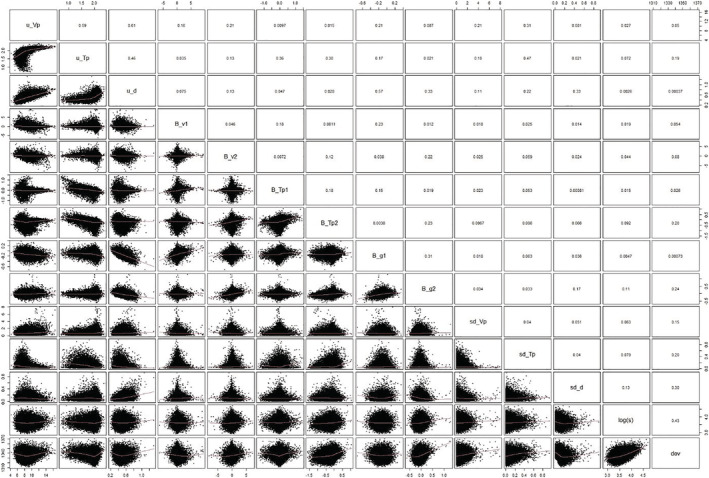
Correlation matrix of pairwise posterior distributions. Upper panels are the Pearson's correlation coefficients

## Data Availability

The data that support the findings of this study are available from the corresponding author upon reasonable request.

## APPENDIX A MODEL SPECIFICATION AND BAYESIAN INFERENCE OF PARAMETERS QUANTIFYING SHEDDING OF AVIAN INFLUENCE VIRUS

We provide full details of the model used to estimate the dynamic shedding of avian infleunza virus (AIV) in challenged captive wild mallards, including the Bayesian framework used to estimate parameters in the model and model fit diagnostics.

Oral virus shedding curves were inferred for intrachoanally inoculated mallards by fitting observed oral AIV detection as measured by qRT‐PCR from oropharyngeal swab and quantified as egg infectious doses (EID) with a phenomenological model in which EIDs rise exponentially after inoculation, reaching a maximum level after which it decays exponentially (Handel et al. 2014; Holder et al., 2011). We modeled the EID response as a lognormally distributed response variable,

$$\log\left(\mu_{i,t}\right)∼\text{lnorm}\left({\mathrm{EID}}_{i,t},\log\left(s.\right)\right)$$

The level of observed virus shedding (EID) by animal *i* at *τ* days postinfection is given by the nonlinear function,

$${\mathrm{EID}}_{i,t}=\frac{2\left(V_{p,i}+\beta_{v,1-2}\right)}{e^{-g_{i}*\left(t-\left(t_{p,i+}\beta_{t_{p},1-2}\right)\right)}+e^{\left(d_{i}+\beta_{d,1-2}\right)*\left(t-\left(t_{p,i+}\beta_{t_{p},1-2}\right)\right)}}$$

*V*_*p*_ was the maximum EID at the infection peak for mallards that were seropositive for AIV exposure on the inoculation day with coefficients *β*_*v*,1 − 2_ estimated for additive effects of seronegative adults (*β*_*v*,1_) and subadults (*β*_*v*,2_); *t*_*p*_ and $\left(\beta_{t_{p},1-2}\right)$ were the estimates of time (days postinfection) of the maximum EID; *g* was the exponential growth rate of viral shedding, *d* and (*β*_*d*,1 − 2_) were the exponential rate of viral shedding decline, and *t* was the time (days postinfection, DPI) of the observed EID response.

Individual variation in shedding was incorporated by allowing the each of the parameters to vary among individuals, such that the parameters were drawn from hierarchical gamma distributions. The gamma distributions were parameterized with global mean and standard deviation formulation of the gamma distribution shape and rate parameters, with the exception of the growth parameter,

$$V_{p,i}∼\text{gamma}\left(\frac{{\mu_{\mathrm{Vp}}}^{2}}{{{\mathrm{sd}}_{\mathrm{Vp}}}^{2}},\frac{\mu_{\mathrm{Vp}}}{{{\mathrm{sd}}_{\mathrm{Vp}}}^{2}}\right).$$

$$T_{p,i}∼\text{gamma}\left(\frac{{\mu_{\mathrm{Tp}}}^{2}}{{{\mathrm{sd}}_{\mathrm{Tp}}}^{2}},\frac{\mu_{\mathrm{Tp}}}{{{\mathrm{sd}}_{\mathrm{Tp}}}^{2}}\right).$$

$$d_{i}∼\text{gamma}\left(\frac{{\mu_{d}}^{2}}{{{\mathrm{sd}}_{d}}^{2}},\frac{\mu_{d}}{{{\mathrm{sd}}_{d}}^{2}}\right).$$

$$g_{i}∼\text{unif}\left(1,10\right).$$

We did not estimate the exponential growth rate parameter, *g*, because it was nonidentifiable given the sampling frequency. Rather, we included as an informative closed‐form prior that was bounded by biologically relevant values (Lemoine, 2019).

The global mean and standard deviations for the nonlinear function parameters, as well as the lognormal standard deviation of the observed EID, were estimated as hyperpriors with weakly informative prior distribution bounded by biological relevant values informed by previous AIV challenge studies in mallards (Arsnoe et al., 2011; Lemoine, 2019; Singleton, 2015),

$$\mu_{\mathrm{Vp}}∼\text{unif}\left(0,30\right);{\mathrm{sd}}_{\mathrm{Vp}}∼\text{unif}\left(0,100\right).$$

$$\mu_{\mathrm{Tp}}∼\text{unif}\left(0,10\right);{\mathrm{sd}}_{\mathrm{Tp}}∼\text{unif}\left(0,100\right).$$

$$\mu_{d}∼\text{unif}\left(0.001,500\right);{\mathrm{sd}}_{d}∼\text{unif}\left(0,100\right).$$

$$\log\left(s\right)∼\text{unif}\left(0.001,100\right).$$

The prior specification of the parameters for additive effects of seronegative status in adults and subadults (*β*_*v*,1 − 2_), $\left(\beta_{t_{p},1-2}\right)$, and (*β*_*d*,1 − 2_) was a noninformative Cauchy distribution, *cauchy(10, 1)*.

Parameter values were estimated using Gibbs sampling implemented in JAGs (Plummer, 2003) and the R package *jagsUI* (Kellner, 2019) using three parallel MCMC chains of 250 000 iterations following a 50 000 iteration burn‐in. Posterior distributions were generated after confirmed convergence of the MCMC chains (Table A1) and examination of parameter correlation (Figure A1) for all parameters and sampled every 20th iteration.

**TABLE A1:** Parameter estimate and convergence diagnostic table

Parameter	Mean	SD	2.5%	Median	97.5%	Rhat	*N*_eff_
*μ*_*Vp*_	8.600	1.517	6.228	8.404	12.147	1.007	564
*μ*_*Tp*_	1.904	0.221	1.248	1.972	2.15	1.011	425
*μ*_*d*_	0.493	0.103	0.32	0.483	0.725	1.003	1117
*β*_*v*,1_	0.157	0.793	−1.189	0.06	2.195	1.000	30 000
*β*_*v*,2_	0.011	0.746	−1.621	0.005	1.61	1.002	1159
*β*_*tp*,1_	−0.037	0.206	−0.491	−0.031	0.368	1.009	2412
*β*_*tp*,2_	−0.237	0.283	−0.971	−0.175	0.191	1.001	2495
*β*_*g*,1_	−0.127	0.098	−0.328	−0.124	0.061	1.000	30 000
*β*_*g*,2_	−0.031	0.123	−0.262	−0.034	0.222	1.000	9996
*sd*_*Vp*_	0.732	0.607	0.027	0.593	2.21	1.002	7477
*sd*_*Tp*_	0.081	0.089	0.002	0.052	0.337	1.002	1876
*sd*_*d*_	0.113	0.075	0.007	0.103	0.287	1.001	13 814
log(*s*)	3.609	0.231	3.191	3.599	4.09	1.001	3537
deviance	1335.415	7.809	1320.791	1335.324	1351.159	1.002	952

*Note*: Mean, standard deviation (SD), 95% credible interval (2.5%, 97.5%), and median of the posterior parameter distributions. Rhat is the Gelman–Rubin scale reduction statistic for the three parallel MCMC chains, and *N*_eff_ is the effective posterior sample size (Gelman et al., 2013).
