# Supplementary material for: Highly pathogenic avian influenza virus H5N2 (clade 2.3.4.4) challenge of mallards age appropriate to the 2015 midwestern poultry outbreak
Source: Influenza Other Respir Viruses. 2021 Jul 29;15(6):767–77. doi: 10.1111/irv.12886 (PMC8542950; doi:10.1111/irv.12886)
Supplement: Supplementary file 1 — Table S1. Avian influenza virus isolates used in hemagglutination inhibition assays. [file IRV-15-767-s001.docx]

Table S1. Avian influenza virus isolates used in hemagglutination inhibition assays.

Virus name Pathotype Lineage^†^ Clade

A/Mallard/Alberta/195/2016 (H1N1) LPAIV NA -

A/Mallard/Alberta/260/2016 (H1N1) LPAIV NA -

A/Spot-billed Duck/Alberta/266/2016 (H1N2) LPAIV NA -

A/Duck/Ukraine/63 (H3N8) LPAIV NA -

A/Mallard/Alberta/362/2017 (H3N8) LPAIV NA -

A/Duck/Czech/56 (H4N6) LPAIV NA -

A/Duck/Alberta/31/2016 (H4N6) LPAIV NA -

A/Ruddy turnstone/DE/244/91 (H5N2) LPAIV NA -

A/Turkey/MA/3740/65 (H6N2) LPAIV NA -

A/Ruddy turnstone/NJ/65/85 (H7N3) LPAIV NA -

A/Turkey/WI/1/66 (H9N2) LPAIV NA -

A/Mallard/Alberta/380/2017 (H11N9) LPAIV NA -

A/Duck/Alberta/60/76 (H12N5) LPAIV NA -

A/Blue-winged teal/Alberta/233/2016 (H12N5) LPAIV NA -

A/Gyrfalcon/WA/41088-6/2014 (H5N8) HPAIV EA/NA 2.3.4.4A

A/Turkey/MN/11668-1/2015 (H5N2) HPAIV EA/NA 2.3.4.4A

^†^Continent of virus origin. NA-North America; EA-Eurasia
