# Supplementary material for: Highly pathogenic avian influenza virus H5N2 (clade 2.3.4.4) challenge of mallards age appropriate to the 2015 midwestern poultry outbreak
Source: Influenza Other Respir Viruses. 2021 Jul 29;15(6):767–77. doi: 10.1111/irv.12886 (PMC8542950; doi:10.1111/irv.12886)
Supplement: Supplementary file 2 — Table S2. Body weights (grams) of highly pathogenic avian influenza virus H5N2 clade 2.3.4.4A challenged mallards [file IRV-15-767-s002.docx]

Table S2. Body weights (grams) of highly pathogenic avian influenza virus H5N2 clade 2.3.4.4A challenged mallards

Day Post-inoculation

| Cage # | Bird ID | Treatment | 0 | 2 | 4 | 6 | 8 | 10 | 12 |
| --- | --- | --- | --- | --- | --- | --- | --- | --- | --- |
| 1 | R45 | Control | 1106 | 1102 | 1124 | 1103 | 1124 | 1107 | 1121 |
|  | Y264B | Control | 1124 | 1025 | 1017 | 981 | 956 | 951 | 966 |
| 2 | R100 | Inoculated | 1024 | 966 | 918 | 939 | 937 | 1001 | 981 |
|  | R395 | Transmission^†^ |  | 1134 | 1124 | 1118 | 1166 | 1177 | 1219 |
| 3 | R66 | Inoculated | 850 | 856 | 910 | 877 | 876 | 864 | 885 |
|  | R186 | Transmission |  | 1082 | 1038 | 995 | 970 | 1075 | 1121 |
| 4 | R41 | Inoculated | 973 | 931 | 912 | 936 | 960 | 982 | 1028 |
|  | R42 | Transmission |  | 926 | 888 | 862 | 899 | 907 | 940 |
| 5 | R269 | Inoculated | 1343 | 1296 | 1250 | 1238 | 1247 | 1242 | 1215 |
|  | R268 | Transmission |  | 1001 | 983 | 1002 | 1002 | 1016 | 997 |
| 6 | Y298 | Inoculated | 957 | 907 | 868 | 882 | 944 | 984 | 983 |
|  | Y189 | Transmission |  | 1106 | 1065 | 1050 | 1085 | 1096 | 1122 |
| 7 | Y285 | Inoculated | 910 | 869 | 884 | 902 | 874 | 896 | 911 |
|  | Y293 | Transmission |  | 1003 | 1002 | 978 | 977 | 991 | 997 |
| 8 | R35 | Inoculated | 936 | 942 | 963 | 950 | 965 | 957 | 955 |
|  | R48 | Transmission |  | 1140 | 1135 | 1180 | 1183 | 1189 | 1197 |
| 9 | Y279 | Inoculated | 931 | 866 | 832 | 823 | 855 | 886 | 892 |
|  | Y271 | Transmission |  | 1088 | 1080 | 1108 | 1192 | 1089 | 1090 |
| 10 | R257 | Inoculated | 1139 | 1107 | 1085 | 1081 | 1128 | 1150 | 1171 |
|  | Y251 | Transmission |  | 815 | 790 | 819 | 863 | 850 | 865 |
| 11 | Y252B | Inoculated | 1196 | 1132 | 1094 | 1104 | 1072^‡^ |  |  |
|  | Y261 | Transmission |  | 930 | 896 | 864 | 938 | 966 | 984 |
| 12 | Y265B | Inoculated | 1012 | 1040 | 994 | 992 | 962 | 944 | 898 |
|  | Y286 | Transmission |  | 978 | 956 | 990 | 920 | 996 | 996 |
| 13 | Y257B | Inoculated | 1212 | 1124 | 1086 | 1050 | 1082 | 1136 | 1168 |
|  | R498 | Transmission |  | 1182 | 1236 | 1218 | 1264 | 1326 | 1346 |
| 14 | R43 | Inoculated | 1052 | 1006 | 978 | 952 | 998 | 1044 | 1036 |
|  | Y262B | Transmission |  | 1208 | 1142 | 1138 | 1230 | 1142 | 1076 |
| 15 | Y297 | Inoculated | 1048 | 994 | 966 | 928 | 970 | 1004 | 1014 |
|  | Y261B | Transmission |  | 1110 | 1068 | 1076 | 1088 | 1084 | 1096 |
| 16 | R46 | Inoculated | 1082 | 1050 | 1004 | 1072 | 1066 | 1088 | 1124 |
|  | Y260B | Transmission |  | 1306 | 1270 | 1209 | 1186 | 1098 | 1190 |
| 17 | R369 | Inoculated | 1166 | 1124 | 1112 | 1144 | 1144 | 1146 | 1150 |
|  | Y259B | Transmission |  | 1159 | 1108 | 1108 | 1088 | 1092 | 1082 |
| 18 | Y290 | Inoculated | 988 | 972 | 994 | 1070 | 1102 | 1120 | 1140 |
|  | Y251B | Transmission |  | 1022 | 1062 | 1056 | 1066 | 1056 | 1064 |
| 19 | Y254B | Inoculated | 1090 | 1036 | 998 | 1004 | 1028 | 1048 | 1048 |
|  | Y256B | Transmission |  | 946 | 920 | 874 | 842 | 872 | 890 |
| 20 | Y258B | Inoculated | 1150 | 1078 | 1032 | 1020 | 1088 | ND | 1112 |
|  | Y263B | Transmission |  | 1078 | 1040 | 1038 | 1082 | ND | 1096 |

^†^ Transmission subjects not weighed on Day 0 ^‡^Bird euthanized DPI 8

Sub-adult birds designated with B in Bird ID
